# Supplementary material for: Molecular detection of dugbe orthonairovirus in cattle and their infesting ticks (Amblyomma and Rhipicephalus (Boophilus)) in Nigeria
Source: PLoS Negl Trop Dis. 2021 Nov 17;15(11):e0009905. doi: 10.1371/journal.pntd.0009905 (PMC8598060; doi:10.1371/journal.pntd.0009905)
Supplement: Attachment — Submitted filename: Revision.docx [file pntd.0009905.s001.docx]

The following revisions and suggestions should be considered by the authors:

Line 2: “and” was mistakenly italicized.

Line 31: “…….qPCR and positive….” A comma should be inserted before “and”.

Line 29 (and Line 46): “Blood and or attached ticks were collected from 1051 cattle” The ticks were collected from the cattle; therefore, it seems sufficient to simply write "ticks" instead of "attached ticks".

Line 37 (and Line 54): “circulating in Nigeria with an evidence of only slight genetic deviation from the original strain.” It is difficult to argue with certainty whether the virus circulating today has deviated genetically over time, as previous studies have not provided sufficient detail about the virus circulating in the study area. There may be many possibilities, such as the virus detected in this study being a genotype that could not be detected in previous studies and became dominant in the population over time, etc... More detailed data are needed to assert such a deviation of the virus at the entire population level in a given environment in a relatively short time (decades). I think, the difference between the viruses should of course be stated, but related sentences may be modified to avoid possible misunderstanding.

Line 59: “Dugbe orthonairovirus (DUGV) was first isolated in Nigeria out of Amblyomma……” For this sentence, “from Amblyomma” sounds to be better than “out of Amblyomma”. (not mandatory)

Line 66: “(N) respectively [3]”…… “,” before “respectively”

Line 79: “DUGV [18, 19] which has been categorized as a BSL 3 pathogen [9]”. The determination of the biosafety level of an agent can vary by country (regarding endemicity). The country or geographic region may be specified.

Line 114: “were arbitrarily collected”….. “randomly” could be preferred instead of “arbitrarily”

Line 129: “Amblyomma and Rhipicephalus (Boophilus)” Genus names must be written in italics.

Line 178: It may be more appropriate for sentences or paragraphs not to start with numbers.

Line 244: Virus positivity varies according to the tick species. Therefore, this comparison can only make sense through Amblyomma, which has significant positivity. Same sentence with same calculation (%) can be done using the numbers of Amblyomma.

Line 291: Use abbreviation for Rhipicephalus and Boophilus (R. and B.)

Line 296-303: Sampling methodology of this study is insufficient to make a detailed comparison of tick community. The data about the tick populations given in the Results section will be sufficient. These lines should either be deleted or thoroughly summarized.

Line 309-310: Here again, sampling methodology of this study is insufficient to make detailed comments on the seasonality of the virus. Such an inference can only be possible with virus screenings performed on a certain number of ticks collected monthly or at least seasonal basis from the same animal groups managed with same process (grazing etc.) in the same localities.

Line 395, 369, 397, 406, 415, and 437: Unlike other references, all the words in the sentences giving the name of the studies began with capital letters in these references.

Line 366, 412, 443: Genus names must be written in italics.
