# Supplementary material for: Molecular detection of dugbe orthonairovirus in cattle and their infesting ticks (Amblyomma and Rhipicephalus (Boophilus)) in Nigeria
Source: PLoS Negl Trop Dis. 2021 Nov 17;15(11):e0009905. doi: 10.1371/journal.pntd.0009905 (PMC8598060; doi:10.1371/journal.pntd.0009905)
Supplement: Attachment — Submitted filename: Revision Letter to the Editor_Authors response[1].docx [file pntd.0009905.s002.docx]

Dear Editor-in-Chief,

We want to appreciate all the three reviewers of our manuscript for deep reading and valuable contributions.

We have responded to all their comments and we indeed found that that has immensely improved the write up.

Please find below our responses and we hope to hear from you soon.

Thank you and kind regards,

Prof. Dr. M. H. Groschup

**MAIN MANUSCRIRPT COMMENTS**

The following revisions and suggestions should be considered by the authors:

Line 2: “and” was mistakenly italicized.

Response: corrected as proposed.

Line 31: “…….qPCR and positive….” A comma should be inserted before “and”.

Response: corrected as proposed.

Line 29 (and Line 46): “Blood and or attached ticks were collected from 1051 cattle” The ticks were collected from the cattle; therefore, it seems sufficient to simply write "ticks" instead of "attached ticks".

Response: corrected as proposed.

Line 37 (and Line 54): “circulating in Nigeria with an evidence of only slight genetic deviation from the original strain.” It is difficult to argue with certainty whether the virus circulating today has deviated genetically over time, as previous studies have not provided sufficient detail about the virus circulating in the study area. There may be many possibilities, such as the virus detected in this study being a genotype that could not be detected in previous studies and became dominant in the population over time, etc... More detailed data are needed to assert such a deviation of the virus at the entire population level in a given environment in a relatively short time (decades). I think, the difference between the viruses should of course be stated, but related sentences may be modified to avoid possible misunderstanding.

Response: the authors thank the reviewer for his valuable comment. To avoid any ambiguities the sentences have been changed to: “Hence, DUGV with high similarity to the previously characterised strain has been detected in Nigeria.” and “Hence, we conclude that a DUGV strain very similar to the priorly characterised genotype can be found in Nigeria.”

Line 59: “Dugbe orthonairovirus (DUGV) was first isolated in Nigeria out of Amblyomma……” For this sentence, “from Amblyomma” sounds to be better than “out of Amblyomma”. (not mandatory)

Response: corrected as proposed.

Line 66: “(N) respectively [3]”…… “,” before “respectively”

Response: corrected as proposed.

Line 79: “DUGV [18, 19] which has been categorized as a BSL 3 pathogen [9]”. The determination of the biosafety level of an agent can vary by country (regarding endemicity). The country or geographic region may be specified.

Response: Nigeria uses CDC biosafety level classification and that is the reason why we have classified DUGV as so

Line 114: “were arbitrarily collected”….. “randomly” could be preferred instead of “arbitrarily”

Response: the authors used the term “arbitrarily” to outline that no specific selection method was applied. The term “randomly” in the context of epidemiological/epizootiological surveys could be interpreted as there is a specific method of randomification, i.e. by algorithm-based selection for coincidence to exclude human bias. Therefore, we would like to keep this term.

Line 129: “Amblyomma and Rhipicephalus (Boophilus)” Genus names must be written in italics.

Response: corrected as proposed.

Line 178: It may be more appropriate for sentences or paragraphs not to start with numbers.

Response: The sentence was rewritten to avoid numerics at the beginning.

Line 244: Virus positivity varies according to the tick species. Therefore, this comparison can only make sense through Amblyomma, which has significant positivity. Same sentence with same calculation (%) can be done using the numbers of Amblyomma.

Response: This has been adjusted as requested

Line 291: Use abbreviation for Rhipicephalus and Boophilus (R. and B.)

Response: corrected as proposed.

Line 296-303: Sampling methodology of this study is insufficient to make a detailed comparison of tick community. The data about the tick populations given in the Results section will be sufficient. These lines should either be deleted or thoroughly summarized.

Response: comparison has been properly summarized

Line 309-310: Here again, sampling methodology of this study is insufficient to make detailed comments on the seasonality of the virus. Such an inference can only be possible with virus screenings performed on a certain number of ticks collected monthly or at least seasonal basis from the same animal groups managed with same process (grazing etc.) in the same localities.

Response: the authors agree with the notion of the reviewer and mitigated their interpretation of the results accordingly.

Line 395, 369, 397, 406, 415, and 437: Unlike other references, all the words in the sentences giving the name of the studies began with capital letters in these references.

Response: corrected as proposed.

Line 366, 412, 443: Genus names must be written in italics.

Response: corrected as proposed.

**GENERAL COMMENTS**

Reviewer #1: -	196bp is pretty a quite small fragment to do phylogeny. I appreciate that some Nanopore sequencing was performed, but only for one sample. If the qPCR primers amplify a very conserved region, it’s actually very hard to conclude that all your samples are closely related to the IbAr 1792 strain. And of course, no conclusion can be drawn about the diversity of the strains based on Nanopore sequencing since only one sample was used. Whether the strains are all similarly closely related to IbAr 1792 (as you said) or there is diversity in your samples, both scenarios are interesting, but needs to be better supported. Not all samples need to be sequenced but I would like to see more sequences of adequate size for reliable phylogeny, ideally from very different samples.

Response: Thank you for your comment. We strongly agree that more sequences would have improved the content of this manuscript. Unfortunately, the quality of only one sample was sufficient to sequence larger segments. In general, it may have been because the ticks were stored in ethanol for a relatively long time (over 1 year) until processing could continue. Therefore, we are very happy to have received a complete S segment at all. We hope that you can understand our situation and we want to let you know that we are annoyed ourselves that we could not generate longer sequences for the other samples.

-	The authors mention that ticks were placed in 2ml Eppendorf tubes. Engorged Amblyomma ticks get pretty big, too big to fit in those specific tubes. Did they authors pick partially engorged Amblyomma ticks on purpose?

Response: Only those Amblyomma ticks which were of appropriate size were used for further analysis. That means completely engorged female Amblyomma were excluded. However, only a very small fraction of the ticks collected were of such size. Thus, the following sentence were added: “Fully engorged *Amblyomma* ticks which did not fit into the tube were excluded.”

-	Please indicate the number of the protocol approved by the Ethics committee for this work. Also clarify who performed the animal blood draws.

Response: this information has been included

**Results**

Reviewer #1: - The authors used the term "frequency" to report the number of ticks collected. Frequency is by definition a number of occurrences PER UNIT OF TIME. It is not appropriate here.

- Please indicate the range of CT values of the samples selected for sequencing

Response: the term “frequency” was changed to “Tick count” and the Ct-Values of the selected samples for RT-qPCR have been added.

**Conclusions**

Reviewer #1: -	It is not “extremely difficult to draw definite conclusions on vector competence based on this data”, it is impossible. Your data contributes to what is known about ticks harbouring DUGV, but there’s no way we can extrapolate on vector competence.

Response: the authors agree with the reviewer that for any statements about vector competence other studies are necessary. Thus we changed the phrase from “extremely difficult” to “impossible”. In fact, those vector competence experiments have been conducted for *Ambylomma variegatum*, which have been also cited in the manuscript:

- Booth TF, Marriott AC, Steele GM, Nuttall PA. Dugbe virus in ticks: histological localization studies using light and electron microscopy. Arch Virol 1990);1: 207-218. doi: 10.1007/978-3-7091-9091-3_23
- Cornet JP, Georges AJ, Gonzalez JP. Transovarian transmission of Dugbe virus in *Amblyomma variegatum* ticks. Annales de l'Institut Pasteur / Virologie 1987;138 269-271. doi:10.1016/S0769-2617(87)80011-4

**Editorial and Data Presentation Modifications?**

Reviewer #1: -	The running title is missing

-	The author’s summary is a copy/paste from the abstract with a few scientific terms replaced. This is not what an Author’s summary is. Please look again at the guidelines.

Response: both a running title was added and the author’s summary reformulated to fit according to the journal guidelines.

-	I would remove the estimates of ticks per cattle, simply obtained by calculating an average. When several hosts are available, ticks never infest them homogeneously. I understand that this value is sometimes helpful to compare burdens, but maybe only compare raw ratio instead of stating that “on average x ticks were found on each animal”.

Response: the estimate has been removed. Differences are now based on percentage

**Summary and General Comments**

Reviewer #1: This manuscript by Daodu et al. provides new surveillance data on the circulation of Dugbe virus. After being first detected in Nigeria, it is very interesting to see more recent data. The manuscript is overall well written, especially the discussion section that I found of very good quality. The reason I am requesting major revision is because the authors are concluding that the strains they detected are all closely related to IbAr 1792, based on 196bp amplicons. I would like to see more sequences.

Response: We could only sequenced one out of the DUGV-positive samples (best quality) as earlier explained. However, the M-segment and L-segment of this best quality DUGV-positive sample were sequenced and have been added to the manuscript. The S-, M- and L-segment sequences were closely related to each of the segments of IbAr 1792 strain.

Reviewer #2: (No Response)

Reviewer #3: Overall, the study presents updated prevalence data of circulating Dugbe virus from cattle ticks. Given phylogenetic sequence analysis for the S-segment, very little variance has occurred in this virus. It would have beneficial if M-segment phylogeny work had been done to assess if the virus had undergone any reassortment events these last forty years from the previous work in the region.

Response: As already noted above, the sample with the complete S-segment is already the one with the best quality. However, we have considered the comment regarding the M and L segments and have actually been able to present complete M and L segment of the same sample. Accordingly, we built two new phylogenetic tree for these segment (in addition to the initial tree) and modified the text. We hope that this has improved the quality of the paper and thank the reviewers for this valuable advice.
